# Structural Characterization of *Pandoraea pnomenusa* B-356 Biphenyl Dioxygenase Reveals Features of Potent Polychlorinated Biphenyl-Degrading Enzymes

**DOI:** 10.1371/journal.pone.0052550

**Published:** 2013-01-03

**Authors:** Christopher L. Colbert, Nathalie Y. R. Agar, Pravindra Kumar, Mathew N. Chakko, Sangita C. Sinha, Justin B. Powlowski, Lindsay D. Eltis, Jeffrey T. Bolin

**Affiliations:** 1 Department of Chemistry and Biochemistry, North Dakota State University, Fargo, North Dakota, United States of America; 2 Department of Neurosurgery and Radiology, Brigham and Women's Hospital, Harvard Medical School, Boston, Massachusetts, United States of America; 3 Department of Biotechnology, Indian Institute of Technology, Roorkee, Uttarakhand, India; 4 Department of Diagnostic Radiology, Providence Hospital and Medical Centers, Southfield, Michigan, United States of America; 5 Department of Chemistry and Biochemistry, Concordia University, Montreal, Quebec, Canada; 6 Departments of Microbiology and Biochemistry, Life Sciences Institute, University of British Columbia, Vancouver, Canada; 7 Department of Biological Sciences and Center for Cancer Research, Purdue University, West Lafayette, Indiana, United States of America; University of Graz, Austria

## Abstract

The oxidative degradation of biphenyl and polychlorinated biphenyls (PCBs) is initiated in *Pandoraea pnomenusa* B-356 by biphenyl dioxygenase (BPDO_B356_). BPDO_B356_, a heterohexameric (αβ)_3_ Rieske oxygenase (RO), catalyzes the insertion of dioxygen with stereo- and regioselectivity at the 2,3-carbons of biphenyl, and can transform a broad spectrum of PCB congeners. Here we present the X-ray crystal structures of BPDO_B356_ with and without its substrate biphenyl 1.6-Å resolution for both structures. In both cases, the Fe(II) has five ligands in a square pyramidal configuration: H233 Nε2, H239 Nε2, D386 Oδ1 and Oδ2, and a single water molecule. Analysis of the active sites of BPDO_B356_ and related ROs revealed structural features that likely contribute to the superior PCB-degrading ability of certain BPDOs. First, the active site cavity readily accommodates biphenyl with minimal conformational rearrangement. Second, M231 was predicted to sterically interfere with binding of some PCBs, and substitution of this residue yielded variants that transform 2,2′-dichlorobiphenyl more effectively. Third, in addition to the volume and shape of the active site, residues at the active site entrance also apparently influence substrate preference. Finally, comparison of the conformation of the active site entrance loop among ROs provides a basis for a structure-based classification consistent with a phylogeny derived from amino acid sequence alignments.

## Introduction

Polychlorinated biphenyls (PCBs) are among the most pervasive and persistent chlorinated environmental pollutants despite long-term regulation of their manufacture and use [1]. The discovery that many bacterial strains are able to at least partially degrade PCBs has fueled research directed toward improving bioremediation strategies to clean-up contaminated sites. In aerobic bacteria, PCBs are degraded co-metabolically by enzymes of the biphenyl (Bph) pathway [2]. The first four Bph enzymes comprise a typical *meta*-cleavage pathway involving the initial generation and subsequent ring fission of a catecholic metabolite. Bacterial strains vary widely in their abilities to degrade PCBs. However, the most potent PCB-degrading organisms, exemplified by *Burkholderia xenovorans* LB400 [3], *Rhodococcus jostii* RHA1 [4] and *Pandoraea pnomenusa* B-356 (formerly *Comomonas testosteroni* B-356 [5]), are able to transform congeners containing up to 7 chloro substituents.

**Figure 1:**
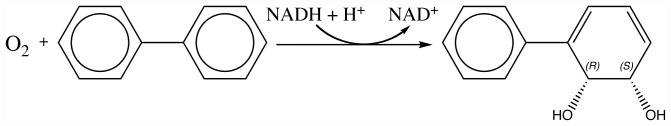
Scheme showing the initial reaction in the Bph pathway.

Biphenyl dioxygenase is the first enzyme of the Bph pathway and the major determinant of PCB degradation [2]. Indeed, the reported PCB-degrading abilities of bacterial isolates largely reflect the PCB-transforming potency of their biphenyl dioxygenase. The catalytic component of this enzyme is a Rieske-type oxygenase (RO), which catalyzes the highly regio- and stereoselective insertion of dioxygen into an aromatic ring, activating the latter for subsequent catabolism. In addition to the oxygenase component (BphAE or BPDO), biphenyl dioxygenase incorporates an FAD-containing reductase (BphG) and a “Rieske-type” ferredoxin (BphF). With biphenyl as substrate, the reaction product is phenyl-(1*R*,2*S*)-cyclohexa-3,4-dienediol or 2,3-dihydroxy-biphenyldiol (Figure 1).

Structural analyses of several ROs, including those of naphthalene dioxygenase (NDO_9816-4_) [6], BPDO_RHA1_ from *R. jostii* RHA1 [7], and BPDO_B1_ from *Sphingobium yanoikuyae* B1 [8] have provided important insights into the architecture and reactivity of PCB-transforming enzymes. It should be noted that BPDO_B1_ degrades naphthalene and phenanthrene in addition to biphenyl [9] and the PCB-degrading properties of *R. jostii* RHA1 have recently been attributed to another RO produced by the organism [10]. These ROs are heterohexameric proteins consisting of α- and β-subunits. The α-subunits contain two domains: a Rieske ferredoxin domain and a mononuclear Fe(II) catalytic domain. Overall, the αβ protomers are arranged around a 3-fold axis with the α-subunits stacked with the β-subunits. A functionally important consequence of this arrangement is that it places the Rieske domain from one α-subunit against the Fe(II) catalytic domain of an adjacent α-subunit.

Potent PCB-degrading BPDOs have been classified into two types based on their congener preference [11]-[13]. KF707-type BPDOs preferentially transform double *para*-substituted congeners, such as 4,4′-dichlorobiphenyl, while LB400-type enzymes preferentially transform *ortho-* and *meta-*substituted congeners [14]–[16]. BPDO_LB400_ is further distinguished by its ability to dehalogenate some *ortho*-substituted congeners, and to catalyze the 3,4-dihydroxylation of others, such as 2,5,2′,5′-tetrachlorobiphenyl.

Mondello and coworkers identified four regions in the α-subunit catalytic domain corresponding to BPDO_LB400_ residues 239–239, 277, 335–341, and 379 that were proposed to confer substrate specificity for the KF707-type and LB400-type BPDOs [15]. BPDO_B356_ from *P. pnomenusa* B-356 would be classified as a KF707-type enzyme based on the analysis of its sequence within these regions. However, BPDO_B356_ preferentially transforms *ortho-* and *meta-*substituted congeners [17]. More recent studies have revealed that BPDO_B356_ appears to be an even more potent PCB-degrading enzyme than BPDO_LB400_, and catalyzes the 3,4-dihydroxylation of some congeners [18]. Structural data are clearly required to properly understand the determinants of congener preference and regioselectivity of BPDOs. Moreover, such data will facilitate the directed evolution and protein engineering efforts to augment the PCB-transforming potency of these enzymes [19]–[21].

We report herein the X-ray crystal structure of BPDO_B356_ at 1.5 Å resolution, as well as the 1.6 Å structure of the BPDO_B356_:biphenyl binary complex. Detailed structural analyses in context of the other known RO structures enabled us to identify structural determinants of congener selectivity. One of these determinants was verified using directed mutagenesis to generate a variant BPDO whose activity against biphenyl and 2,2′-dichlorobiphenyl was tested. Overall, these studies provide a structure-based rationale for the PCB-degrading abilities of BPDOs facilitating the further engineering of these enzymes.

## Results

### Crystallization of BPDO_B356_

BPDO_B356_ crystals were grown in an anaerobic environment (≤2 ppm O_2_) to maintain the oxidation state of the iron centers. The characteristically reddish-brown colored crystals exhibited a rhombic morphology and belonged to the space group *R*3. The asymmetric unit contains one αβ protomer (V_m_ = 2.7 Da/Å^3^) and the best crystals diffracted to 1.5 Å resolution.

### Crystallographic refinement and final model

The final models include the complete β-subunit, but lack the 18 N-terminal residues of the α-subunit, which were never represented in the electron density and were assumed to be present and disordered. All structures were refined to between 1.5 and 1.6 Å resolution with final *R*_work_ and *R*_free_ values less than 20%. Additional data and statistics are presented in Table 1.

**Table 1:** Refinement parameters and statistics.

Model Content (non-hydrogen atoms)	BPDO_B356_ MES buffer	BPDO_B356_•biphenyl MES buffer
Protein atoms	5015	5015
protein atoms in alternate conformations	105	80
Fe(III), Fe(II) & S^2−^ atoms	5	5
water oxygen atoms	582	640
Diffraction Data		
resolution range (Å)	10-1.63	80-1.58
number of reflections	86168	91502
twin fraction (α)	0.36	0.02
R_refined_ (%)	11.7	17.8
R_free_ (%)	16.6	19.8
Average *B* values (Å^2^)		
protein atoms (main, side chain)	12.1, 15.6	22.4, 22.6
Fe(III), Fe(II), & S^2−^	10.5, 17.8, 11.0	17.8, 26.8, 18.5
water oxygen atoms		32.5
all atoms		24.7
Rms deviations from restraints		
bond lengths (Å)	0.016	0.008
bond angles (°)	1.72	1.12

### Quaternary Structure of BPDO and Phylogenetic Analysis

**Figure 2:**
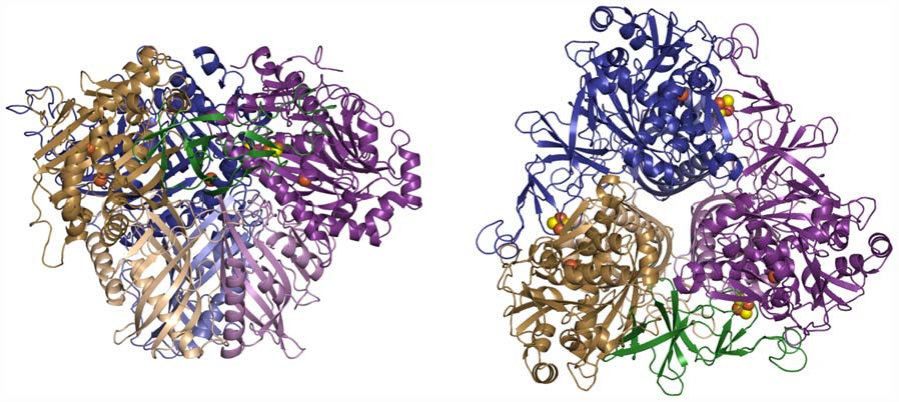
Ribbon diagram showing the overall structure of BPDO_B356_. Two orthogonal views showing three αβ protomers arranged around the crystallographic three-fold axis to form the active hexamer. This arrangement allows the Rieske domain (green ribbons) from the tan α-subunit to interact with the catalytic Fe^2+^(rust sphere) in the adjacent subunit (purple ribbons). All structural graphics were created using Pymol (www.pymol.org).

BPDO_B356_ is an (αβ)_3_ heterohexamer, which is similar to previously reported structures of ROs, such as NDO_9816-4_ [6] (Figure 2). The heterohexameric ROs have very similar three-dimensional structures with rmsds between 0.7–2.4 Å for the Cα atoms of the α-subunits and between 0.7–1.3 Å for the β-subunits.

**Figure 3:**
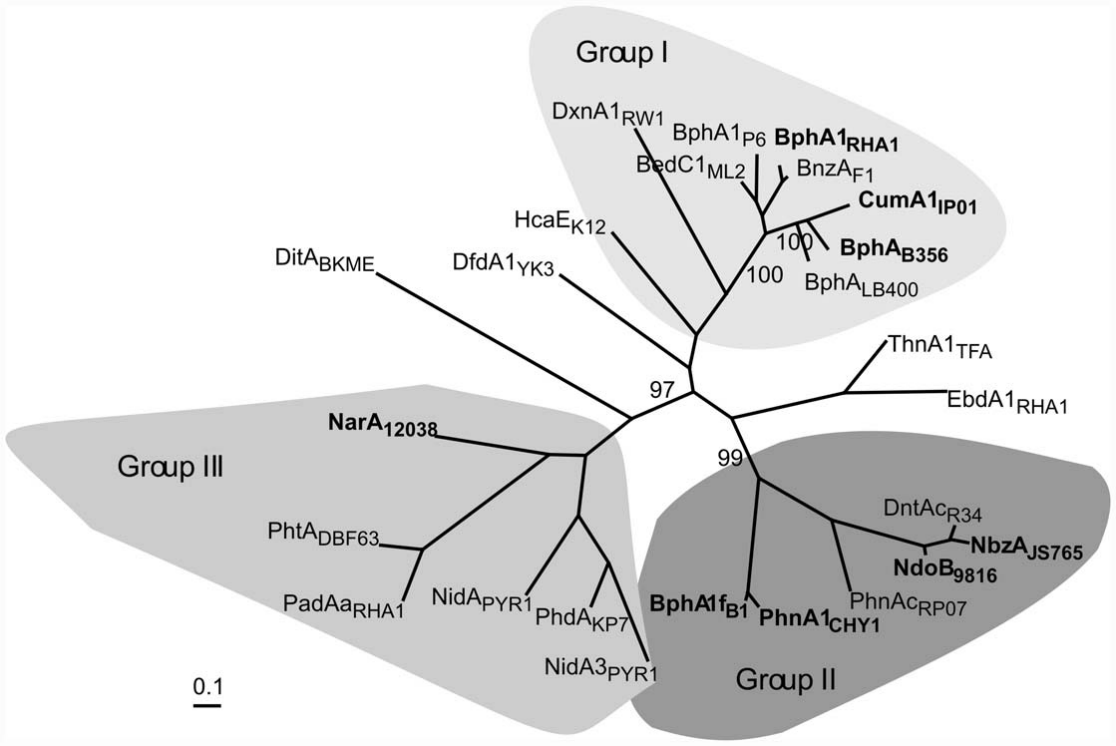
Unrooted phylogenetic tree obtained from a crystal structure-based sequence alignment of 25 α-subunits of related Rieske Oxygenases. Bootstrap values out of 100 replicates are indicated. The proteins are abbreviated using the gene name and strain as follows: biphenyl dioxygenase from *Pandoraea pnomenusa* B-356 (BphA_B356_, GenPeptID: AAC44526), *Burkholderia xenovorans* LB400 (BphA_LB400_, GenPeptID: YP_556409), *Rhodococcus globerulus* P6 (BphA1_P6_, GenPeptID: CAA56346), *Rhodococcus jostii* RHA1 (BphA1_RHA1_, GenPeptID: BAA06868), and *Sphingobium yanoikuyae* B1 (BphA1f_B1_, GenPeptID: ABM91740); benzene dioxygenase from *Pseudomonas putida* ML2 (BedC1_ML2_, GenPeptID: Q07944); benzoate dioxygenase from *P. putida* F1 (BnzA_F1_, GenPeptID: A5W4F2); cumene dioxygenase from *Pseudomonas fluorescens* IP01 (CumA1_IP01_, GenPeptID: BAA07074); dibenzofuran dioxygenase from *Terrabacter* sp. YK3 (DfdA1_YK3_, GenPeptID: BAC06602); diterpenoid dioxygenase from *Pseudomonas abietaniphila* BKME (DitA_BKME_, GenPeptID: AAD21063); dinitrotoluene dioxygenase from *Burkholderia cepacia* R34 (DntAc_R34_, GenPeptID: AAL50021); dibenzo-*p*-dioxin dioxygenase from *Sphignomonas* (DxnA1_RW1_, GenPeptID: CAA51365); ethylbenzene dioxygenase from *R jostii* RHA1 (EbdA1_RHA1_, GenPeptID: BAC92718); 3-phenylpropionate dioxygenase from *E. coli* K-12 (HcaE_K12_, GenPeptID: ACB03690); naphthalene dioxygenase from *Rhodococcus* sp. NCIMB 12038 (NarA_12038_, GenPeptID: AAD28100) and *Pseudomonas putida* 9816-4 (NdoB_9816_, GenPeptID: P0A110); nitrobenzene dioxygenase from *Comomonas* sp. JS765 (NbzA _JS765_, GenPeptID: AAL76202); polyaromatic hydrocarbon dioxygenase from *Mycobacterium vanbaalenii* PYR-1(NidA_PYR1_, GenPeptID: AAF75991, NidA3_PYR1_, GenPeptID: AAY85176) and *Burkholderia* sp. RP007 (PhnAc_RP07_, GenPeptID: AAD09872); phthalate dioxygenase from *R. jostii* RHA1 (PadAa_RHA1_, GenPeptID: ABG99212) and *Terrabacter* sp. DBF63 (PhtA_DBF63_, GenPeptID: BAC54156); phenanthrene dioxygenase from *Nocardioides* sp. KP7 (PhdA_KP7_, GenPeptID: BAA94708) and *Sphingomonas* sp. CHY1 (PhnA1_CHY1_, GenPeptID: CAG17576); and tetraline dioxygenase from *Sphingomonas macrogoltabidus* TFA (ThnA1_TFA_, GenPeptID: AAN26443). Proteins for which the crystal structure was used for alignment are indicated in bold text in the figure.

The superposition of eight α-subunit crystal structures was used to generate a structure-based alignment profile used to guide the overall alignment of amino acid sequences for 25 homologous ROs. The phylogenetic tree generated using this alignment displays three distinct groups (Figure 3). The available functional data indicate that these groups reflect the substrate specificities of the ROs. For example, Group II contains ROs responsible for hydroxylating nitro-containing aromatics, such as NbzA_JS765_ [22] and Group III contains ROs responsible for hydroxylating phthalate, such as PhtA_DBF63_ [23]. In Group I, the potent PCB degrading enzymes BPDO_B356_ and BPDO_LB400_ cluster together with cumene dioxygenase_IP01_ (CumDO_IP01_) and are distinct from the cluster of benzene dioxygenases that include BPDO_RHA1_. Our revised classification based on crystal structure-based sequence alignments and reliance on functional data, while similar to the scheme developed by Nam and coworkers, which was based only on sequences [24], adds a new group to their classification. Whereas our Group I corresponds quite well with their Group IV, our classification divides their Group III into two groups presented as Group II and Group III in Figure 3. More recently, Kweon *et al.* reported an inclusive classification of Rieske oxygenase systems driven by primary sequence data and encompassing all protein components involved in electron delivery and catalysis [25]. Although the present approach and that used by Kweon *et al.* are distinctly different, the molecular phylogenies conform: Group I in Figure 3 maps to Type IV of Kweon *et al.*, Group II maps to Type III, and Group III maps to Type V.

### Structure of the β-subunit

Despite the global similarity of the known β-subunit structures, as demonstrated by the overall low rmsds of 0.7–1.3 Å (Cα atoms), distinctive structural features divide the structures into two categories, similar to those found based on the phylogenetic analysis of the α-subunit: those that resemble NDO_9816-4_ (Group II) and those that resemble BPDO_B356_ (Group I). The fold and interactions of the N-terminal residues with a neighboring β-subunit constitute one of the differentiating features. In NDO, these residues form a two-turn α helix, which interacts with helix 2_β_ (α2_β_) and α3_β_ of an adjacent β-subunit (Note: Elements of secondary structure are numbered sequentially with separate lists for helices and β strands in each subunit. Subscripts α and β identify the subunit: α2_β_ is the second helix along the chain of the β-subunit. Residues are identified by the one–letter amino acid code with the residue number appended; when necessary, the subunit is indicated by a subscripted α or β). In BPDO_B356_, the observed residues meander across the outer surface of the central sheet of the neighboring β-subunit, interacting with and covering residues that are solvent-exposed in NDO.

Other differentiating features occur in loops that interact with the α-subunit. The loop connecting strands β1_β_ and β2_β_ packs more extensively against the Rieske domain in NDO_9816-4_ than in BPDO_B356_. There is also variation in the loop connecting β5_β_ and β6_β_, which bends towards α1_β_ in BPDO_B356_ and away from it in NDO_9816-4_. The structures of the β-subunits of BPDO_RHA1_ and CumDO_IP01_ are more similar to BPDO_B356_, while that of nitrobenzene dioxygenase_JS765_ (NBDO_JS765_), polyaromatic hydrocarbon ring-hydroxylating dioxygenase_CHY-1_ (RHDO_CHY-1_), BPDO_B1_, and NDO_12308_ are more similar to NDO_9816-4_, which is consistent with sequence-based phylogeny based solely on the α-subunit (Figure 3).

The BPDO_B356_ β-subunit α3_β_-β3_β_ loop participates in a web of hydrogen bonds with side chains from α11_α_ including Q384_α_ and D385_α_, the residues immediately preceding the active site Fe ligand D386_α_. These interfacial interactions may couple α and β in a way that directly affects the ability of the active site to adjust to different substrates. Compared to BPDO_B356_, the corresponding loop in NDO_9816-4_ is approximately 3.0 Å further away from the α-subunit, is not involved in a similarly extensive hydrogen-bonding network, and might not be expected to exert a similar influence on the adaptations of the active site during catalysis. Thus, variations in interactions at this interface could explain previously reported inconsistencies in substrate-profiling experiments based on limited mutagenesis or subunit exchange to probe the role of the β-subunit.

Several studies of α_i_-β_j_ chimeric enzymes have established that the β-subunit plays a role in determining substrate specificity in ROs [26]–[28]. In studies of the BPDOα_LB400_-β_B356_ and BPDOα_B356_-β_LB400_ chimeras, exchange of the β-subunit resulted in an extended substrate range relative to the parental proteins and/or a shift in substrate preference correlated with the source of the β-subunit [27]. Nevertheless, such results are not universal: a chimeric naphthalene-2,4-dinitrotoluene dioxygenase (NDOα_9816-4_-DNTDOβ_DNT_) had no significant change in substrate preference [29].

*Structure of the α-Subunit -* The α-subunit of BPDO_B356_ has two domains: a smaller Rieske ferredoxin domain and a larger mononuclear Fe(II)-containing catalytic domain, in agreement with other characterized ROs. The assembly of the oligomer places the Rieske Fe_2_S_2_ cluster of each subunit near the Fe(II) site of an adjacent subunit. Thus, each α-subunit interacts with an adjacent α-subunit by extending its Rieske domain onto the neighboring α-subunit with the Rieske Fe_2_S_2_ cluster serving as an electron donor during the catalytic cycle (Figure 2).

In BPDO_B356_, the Fe-S cluster is linked to the mononuclear Fe(II) by a hydrogen bonding network comprised of cluster ligand H123 of one α-subunit through D230 to H233 of the adjacent α-subunit to span the 11.4 Å between the two centers. This connection is well conserved in all available RO structures. Disruption of this electron transfer pathway by mutagenesis of the intermediate aspartate resulted in an inactive enzyme [30] (N. Agar, Personal Communication).

Surface features of the α-subunit catalytic domains are more variable than those of the α-subunit Rieske domains or the β-subunits, and hexameric RO structures can be subdivided into two groups based on these variable regions, with NDO_9816-4_, NBDO_JS765_, RHDO_CHY-1_, and BPDO_B1_ clustered in one group, and BPDO_B356_, BPDO_RHA1_ and CumDO_IP01_ constituting the other. The two groups correspond to branches on the phylogenetic tree (Figure 3), even though the structures of the catalytic domains are quite similar with catalytically important residues conserved in structurally equivalent positions.

The most dramatic of the structural variations involves residues of low sequence identity (Figure S1) corresponding to 249–262 in BPDO_B356_, which form the entrance to the active site. Other differences are found in the extended helix (α11_α_) and the C-terminal region, where NDO_9816-4_, NBDO_JS765_, RHDO_CHY-1_, and BPDO_B1_ have an extended helical tail.

### Coordination of the Mononuclear Fe

**Figure 4:**
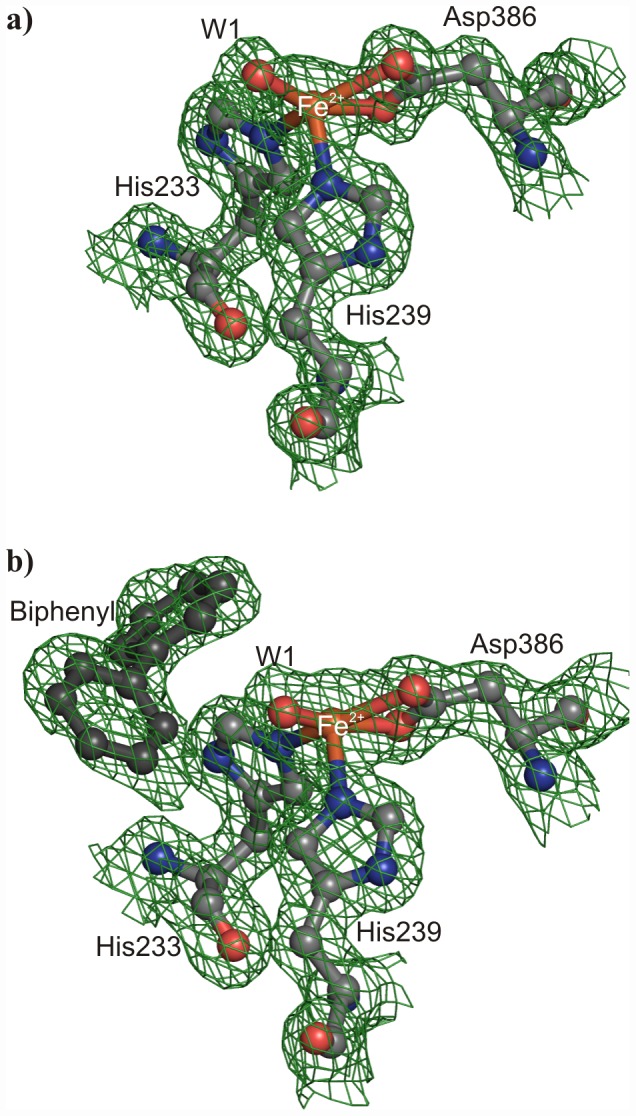
2F_o_-F_c_ electron density maps (contoured at 1σ above the mean) showing the coordination geometry of the mononuclear Fe^2+^. a) The mononuclear Fe^2+^ geometry found in the crystal structure determined in the presence of MES buffer, and b) in the presence of MES buffer and biphenyl. The Fe atom is coordinated by two histidines, a bidentate aspartate, and one water molecule (W1).

BPDO_B356_ was purified anaerobically with Fe(II) in the mononuclear Fe site from the addition of (NH_4_)_2_Fe(SO_4_)_2_·6H_2_O during purification and crystallogenesis with the crystals being flash-frozen while maintaining anaerobic conditions [17]. Previously, this as isolated BPDO_B356_ was determined to have an oxidized Rieske cluster [17]. Further, X-ray absorption scans acquired in association with the diffraction experiments indicated an oxidized Rieske cluster prior to data collection, and qualitatively demonstrated X-ray induced reduction after data collection (data not shown). The as isolated mononuclear Fe site in BPDO_B356_ exhibits square pyramidal coordination by two histidines, a bidentate aspartate, and a water molecule. The BPDO_B356_ structures demonstrate 2.2 Å bonds to the Nε2 of both H233 and H239, as well as bidentate binding to Oδ1 (2.2 Å) and Oδ2 (2.4 Å) of D386 (Figure 4). A single water ligand (W1) at a distance of 2.0 Å is observed (Figure 4a).

The coordination sphere of the mononuclear iron is remarkably similar in the BPDO_B356_:biphenyl complex. As in the substrate-free enzyme, the iron is pentacoordinate and of square pyramidal geometry (Figure 4b). H239 is the axial ligand, and the Fe is displaced toward it out of the basal plane by 0.5 Å. The Fe(II) coordination and geometry is thus similar to a variety of non-RO enzymes that coordinate Fe with histidines and carboxylic acids [31]–[34].

### Structural Influences on Substrate Preference

**Figure 5:**
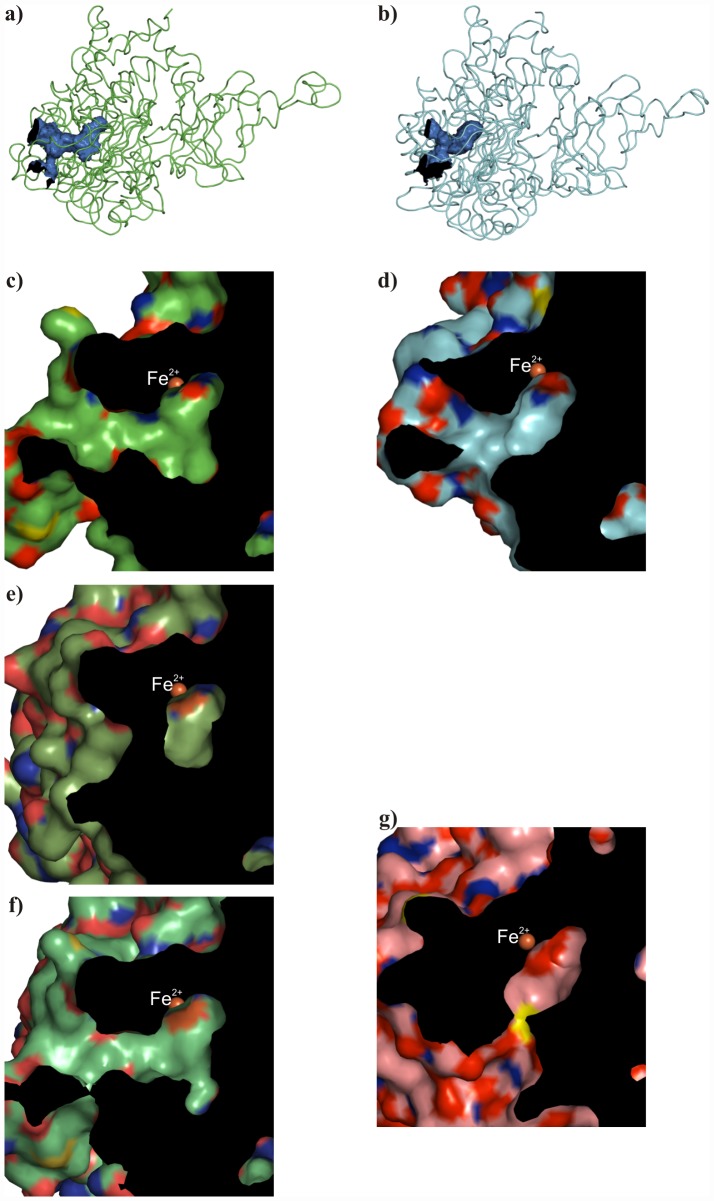
The positions and surface representations of the active site invaginations of BPDO_B356_, NDO_9816-4_, BPDO_RHA1_, CumDO_IP01_, and NDO_12038_. a) Shows the overall active site cavity of BPDO_B356_ as determined by the program VOIDOO. b) The overall active site cavity of NDO_9816-4_ determined similar to a). The solvent accessible surface representations calculated by the program Pymol for c) BPDO_B356_, d) NDO_9816-4_, e) BPDO_RHA1_, f) CumDO_IP01_, and g) NDO_12038_. BPDO_B356_ has a much larger active site cavity relative to BPDO_RHA1_. The distal pocket of CumDO_IP01_ is less pronounced than that of BPDO_B356_. The view of the active site cavity of d) NDO_9816-4_ and g) NDO_12038_ has been rotated slightly relative to that of BPDO_B356_ in order to provide an unobstructed view of the entrance passageway.

The structural analyses suggest that differences both at the entrance to and within the active site cavity of the α-subunit likely contribute to differences in substrate preferences among ROs. In BPDO_B356_, access to the mononuclear Fe is via a 20 Å L-shaped tunnel (Figure 5a,c). This entrance is formed by α6_α_ residues 235-237, α7_α_ residue 240, α7_α_-β17_α_ loop residues 253–259_α_, and β24_α_-α13_α_ loop residue 431_α_.

Residues analogous to the BPDO_B356_ loop residues 253–259_α_ are key components of the active site entrance in all ROs. The location and form of key entrance loop residues are similar for BPDO_B356_ and CumDO_IP01_, and both correspond to the “loop 1” conformation defined for CumDO_IP01_ [35]. In contrast, for the ROs clustered with NDO_9816-4_ [6], the equivalent loop residues have a very different conformation, called “loop 2” by Dong and co-workers [35], where the loop is located at the opposite side of the active site entrance. Thus, different phylogenetic clusters of ROs may use corresponding loop residues differently to regulate access to the active site. Although the entrance residues are disordered in BPDO_RHA1_, the phylogenetic analysis predicts its association with the “loop 1” cluster, and a surface rendering of BPDO_RHA1_ (Figure 5e) confirms that its entrance and cavity are more similar to BPDO_B356_ (Figure 5c) than NDO_9816-4_ (Figure 5d). In summary, we predict that the entrance loop favors the “loop 1” conformation in BPDO_RHA1_ and in all enzymes within the phylogenetic cluster that includes BPDO_B356_ and CumDO_IP01_.

Comparison of the active site cavities of BPDO_B356_-type (Group I) and NDO_9816-4_-type (Group II) ROs reveals further differences. For both types, the cavity can be divided into two subsites: a proximal (P) subsite, which binds the ring that is subject to hydroxylation, and the distal (D) subsite, which binds the second ring of biphenyl in the case of BPDO. For BPDO_B356_, the P subsite is lined by side chains of Q226, F227, H233, H321, L331, and the carbonyl of D230; whereas the distal pocket is lined by residues M231, A234, H239, F277, I283, V287, G319, I334, F376, and F382. Amongst the BPDO_B356_ cluster of ROs, residues lining the P subsite are invariant, while there are only conservative substitutions among the residues lining the D subsite. F277, I283, V287, G319, and F382 in BPDO_B356_ correspond to, F278, L284, I288, A321, and Y384 in CumDO_IP01_ and to Y268, L274, I278, A311, and F374 in BPDO_RHA1_.

With respect to three-dimensional structure, the active site cavities of the various ROs might be compared by semi-quantitative measurements of volume or by assessment of shapes and surface features; the latter appearing seems to be the most revealing approach. For example, Figure 5 compares surface and volume renderings of the active site cavities of BPDO_B356_ (Figure 5c) and NDO_9816-4_ (Figure 5d) and reveals that the cavity of BPDO_B356_ is distinctly bicornuate, whereas that of NDO_9816-4_ appears unicornuate and lacks free space distal to the Fe; thus the NDO_9816-4_ cavity appears relatively flat, consistent with the shape of naphthalene.

For other ROs, the planarity or non-planarity of the substrates is consistent with the architecture of their active sites. The active site of NBDO_JS765_ is similar to NDO_9816-4_ and accommodates a planar substrate. On the other hand the active site architecture of CumDO_IP01_ (Figure 5f), which presumably catalyzes the oxidation of cumene, a molecule distinctly nonplanar although similar in size to nitrobenzene, is similar to that of BPDO_B356_.

A further comparison of the active sites of BPDO_B356_ and CumDO_IP01_ is also of interest. The source bacterial strain for CumDO_IP01_ can co-metabolize, but not grow on, biphenyl [36]. This preference for cumene versus biphenyl can be explained by the obstruction of the D subsite by residue A321 of CumDO_IP01_, thereby creating a smaller cavity (Figure 5f) than in BPDO_B356_ (Figure 5c) with its structurally analogous G319.

Comparison of the active sites of BPDO_B356_ and BPDO_RHA1_ in substrate free and biphenyl bound states reveals a difference that is likely to be important in the context of ability to process larger substrates and a wide range of PCBs. In the BPDO_B356_•biphenyl complex, the 2,3-carbons are 4.3 Å from the Fe(II) and the dihedral angle between the two aromatic rings is 112° (Figure 4b). In the BPDO_RHA1_•biphenyl complex the 2,3-carbons are 4.5 Å from the Fe(II) and the dihedral angle is 124° [7]. Thus, within experimental error, the position and conformation of the substrate are not distinguishable and likely represent a productive binding mode. However, the adjustments in protein conformation that accompany binding of biphenyl are much less dramatic in BPDO_B356_ than in BPDO_RHA1_. In particular, upon binding of biphenyl to BPDO_B356_, the side chain torsion angles of I283 in the D subsite adjust slightly to move the Cδ1 atom away from biphenyl. In contrast, upon biphenyl binding in BPDO_RHA1_, the Cα of the analogous residue, L274, shifts about 2 Å to withdraw the side chain from the D subsite. This movement is part of an overall shift of α8_α_ to accommodate biphenyl [7]. A requirement for large conformational changes to bind biphenyl may translate to lower reactivity of BPDO_RHA1_ towards substrates larger than biphenyl. This hypothesis is consistent with the placement of BPDO_RHA1_ in a phylogenetic cluster occupied by ROs characterized as benzene transforming enzymes.

As a corollary, the less–restricted active site cavity of BPDO_B356_ may explain its greater reactivity to a broad spectrum of substituted biphenyls, such as PCB congeners [17], [18]. Moreover, the arrangement of residues and overall dimensions of the BPDO_B356_ active site cavity may provide a structural explanation of why ROs that clustered with BPDO_B356_ include the well-characterized potent PCB degrading enzymes.

### Mutagenesis and Steady-state Kinetics

**Figure 6:**
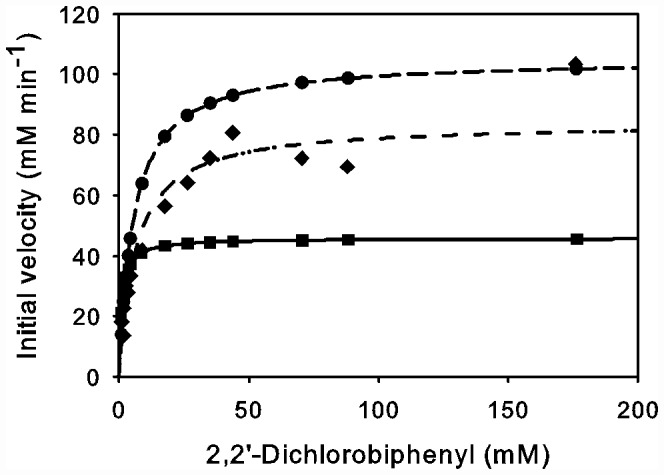
The steady-state dihydroxylation of 2,2′-dichlorobiphenyl by BPDO variants; dependence of the initial velocity of O_2_-uptake on biphenyl concentration in air-saturated buffer. BPDO_B356_ wild-type (▪); BPDO_B356_ M231A (•); BPDO_B356_ M231T (⧫).

The role of the conserved active site residue M231_α_ on the selectivity of BPDO_B356_ was probed by site directed mutagenesis. M231_α_ was chosen because of its unique location at the junction of the P and D subsites and the placement of the M231 Sδ atom in the plane of the proximal ring of biphenyl and near an *ortho* carbon atom on that ring. Substitution of a chlorine at this *ortho* carbon position would result in a stearic clash with the M231 Sδ atom. Therefore, mutations were chosen to alter the steric limitations of the active site (M231A) as well as the polar influences of residues in the active site (M231T). Steady-state kinetic characterizations of these two variants, M231A and M231T, were performed with biphenyl and 2,2′-dichlorobiphenyl substrates (Figure 6). M231A and M231T each showed Michaelis-Menten kinetics for the dependence of the initial rate of oxygen consumption on the concentration of biphenyl. Substituting M231_α_ with either smaller side chain lowered the apparent specificity of the enzyme for both biphenyl (4- to 6-fold) and 2,2′-dichlorobiphenyl (∼3-fold; Table 2). In the event of unproductive catalytic turnover or uncoupling H_2_O_2_ would be generated during the assay and the addition of catalase was used to determine the amount generated. Hydroxylation of biphenyl was well-coupled to O_2_ consumption in the variants. Interestingly, the transformation of the dichlorobiphenyl was better coupled to O_2_ consumption in the variants as compared to wild-type enzyme. In previous studies uncoupling was not detected for wild-type enzyme with biphenyl as substrate [17].

**Table 2:** Apparent steady-state kinetic parameters of BPDO_B356_ wild-type (wt) and variants (M231A and M231T) for biphenyl and 2,2′-dichlorobiphenyl.

	Biphenyl			2,2′-Dichlorobiphenyl			
BPDO_B356_	*K_m_*	*k_cat_*	*k_cat_/K_m_*	*K_m_*	*k_cat_*	*k_cat_/K_m_*	*H_2_O_2_∶O_2_*
	(µM)	(s^−1^)	(×10^6^ M^−1^ s^−1)^	(µM)	(s^−1^)	(×10^6^ M^−1^ s^−1)^	
WT	6.2 (0.5)*	7.3 (0.2)*	1.2 (0.1)*	1.1 (0.2)	1.8 (0.1)	1.7 (0.3)	0.61 (0.03)
M231A	9.4 (1.9)	2.1 (0.1)	0.2 (0.05)	5.7 (0.9)	4.2 (0.2)	0.7 (0.1)	0.42 (0.06)
M231T	11.1 (3.1)	2.9 (0.3)	0.3 (0.1)	6.4 (1.1)	3.4 (0.2)	0.5 (0.1)	0.36 (0.03)

Coupling parameters are given for 2,2′-dichlorobiphenyl only. Standard deviations are given in parenthesis.

* These values were reported in [17].

## Discussion

Here we present the crystal structures of BPDO_B356_ at 1.6 Å resolution, and BPDO_B356_ in complex with its substrate biphenyl at 1.6 Å resolution. BPDO_B356_ is a typical heterohexameric RO with αβ protomers arranged about a three-fold symmetry axis to place the Rieske Fe_2_S_2_ cluster of each α-subunit within ∼12 Å of the mononuclear Fe(II) of an adjacent α-subunit.

The coordination state of the active site Fe has been a significant focus of research on ROs. Prior studies indicate that the redox state of the Rieske center plays an important role in modulating the coordination environment of the mononuclear Fe site. For example, the crystal structure of OxoMO_8_ revealed that the Fe(II) center changed from a pentacoordinate state with a single water ligand to a hexacoordinate state with two water ligands when the crystals were grown in the presence of dithionite [37]. Similarly, side-on binding of O_2_ established a hexacoordinate Fe(II) in NDO_9816-4_ after dithionite reduction of the protein [38]. In contrast, BPDO_B356_ used in the presented study was purified under anaerobic conditions [17], and crystals were subsequently grown and frozen in an anaerobic environment, avoiding the use of strong reducing reagents. Therefore, the observed pentacoordinate state might represent a potential resting state of BPDO_B356_ with Fe(II) in the active site and an oxidized Fe_2_S_2_ cluster. Given the variability of sample treatment and observed coordination states, it remains unclear whether this state is exclusive to BPDO_B356_ or is a common state for all ROs.

Comparison of the active site cavities of BPDO_B356_, CumDO_IP01_, and BPDO_RHA1_ (Group I) to those of BPDO_B1_, NDO_9816-4_, RHDO_CHY-1_ and NBDO_JS765_ (Group II) showed a fundamental distinction between these two groups. Within Group I the active site cavities of BPDO_B356_, CumDO_IP01_, and BPDO_RHA1_ can clearly be subdivided into two distinct subsites based on the invaginations of the active site, the P subsite where ring hydroxylation occurs, and the D subsite that accommodates the rest of the substrate. In contrast, the active site cavities of NDO_9816-4_, NBDO_JS765_, RHDO_CHY-1_, and BPDO_B1_ from Group II contain no such clear divisions.

The structure of BPDO_B356_ may illuminate active site structural factors required for potent PCB-degrading ROs in Group I. Although binding of biphenyl to BPDO_B356_ required only minor adjustments by the protein, biphenyl binding to BPDO_RHA1_ required extensive conformational changes that expand the active site. For CumDO_IP01_, a constriction of the active site cavity due to sequence variation may dictate a preference for cumene over larger potential substrates, such as biphenyl and PCBs. These observations may explain the reactivity of BPDO_B356_ with a broad range of recalcitrant PCB congeners on the basis of facility of aromatic substrate binding alone. By extension, our comparative analysis of these active sites provides a structure-based explanation for the reactivity of related potent PCB degrading ROs such as BPDO_LB400_ and BPDO_KF707_.

For dioxygenases, unhindered binding of the aromatic substrate has determining significance to the binding and activation of the dioxygen substrate. If binding of a particular aromatic substrate challenges the productive binding of dioxygen, the consequence could be increased uncoupling of electron consumption and oxygen activation from the desired reaction. The consequences of a highly uncoupled reaction are highly detrimental and include loss of reducing equivalents with release of reactive oxygen species, inhibition, and suicide inactivation [39].

Finally, as demonstrated by the effect of mutations at M231 on steady state kinetic parameters for the reaction with a representative *ortho*-chlorinated PCB, 2,2′-dichlorobiphenyl, we also showed that strategic alterations of the active site cavity based on the crystal structure can improve the processing of specific PCB congeners. The effects of the M231A and M231T mutations resulted in improvements in turnover number and coupling with the dichloro-substituted substrate, and are consistent with a more accommodating active site. This is further supported by a previous study of BPDO_LB400_, where the corresponding Met to Ala conversion resulted in a variant with significantly altered regioselectivity with two substrates, 2,3′-dichlorobiphenyl and 3,4′dichlorobiphenyl, but the effects on the kinetic parameters and coupling were not reported [40]. Thus, this structural information may contribute to strategies for the engineering of improved bioremediation pathways.

## Materials and Methods

### Phylogenetic Analysis of Rieske Oxygenase Sequences

Sequences used for the phylogenetic analysis were selected from ROs whose X-ray crystal structures have been determined with additional sequences selected from a subset of related sequences. A structure-based sequence alignment was first accomplished by pair-wise superpositions of proteins of known structure. Additional sequences were added and aligned using CLUSTALW [41]. The final alignment was manually adjusted using JalView [42]. This alignment was input into the PHYLIP package [43] and PROML was used to calculate the phylogenetic trees. The best tree was obtained using 21 jumbles of the input alignment. In order to obtain bootstrap values, 100 datasets were generated using SEQBOOT, and then the best tree was calculated from each dataset using three jumbles. The final consensus tree was calculated using CONSENSE.

### Protein Purification and Directed Mutagenesis

BPDO_B356_ and its variants were heterologously produced and purified anaerobically as described previously for the wild-type RO [17]. Directed mutagenesis was performed using the QuikChange protocol (Stratagene) and the following oligonucleotides: 5′-GCAGTTCTGCAGCGA C**GCG**TACCACGCCG-3′ (M231A mutation) and 5′-GCAGTTCTGCAGCGAC**ACG**T ACCACGCCG-3′ (M231T mutation) combined with their reverse complements. *PfuI* DNA polymerase was used amplify the plasmids following annealing of the primers at 52°C.

### Crystallization

Crystals were grown by sitting drop vapor diffusion under anaerobic conditions within a N_2_ atmosphere glove box (Innovative Technologies, Newburyport, MA). Two protocols were used. Crystallization from a solution containing 100 mM sodium citrate, pH 5.8; 10% v/v 2-propanol; and 24% w/v PEG4000 at 20°C was described previously [17]. In the second protocol, protein (36 mg/ml) in 25 mM HEPES, pH 7.3; 2 mM DTT; 10% v/v glycerol; and 0.25 mM ferrous ammonium sulfate was diluted to 7 mg/ml by addition of a solution containing: 25 mM HEPES, pH 7.3; 10% v/v glycerol; 50 mM NaCl; and 0.25 mM ferrous ammonium sulfate. Crystals were obtained via sitting drop vapor diffusion methods by mixing 4 µl of protein solution with 4 µl of a reservoir solution containing: 100 mM MES, pH 6.0; PEG 4000 (18–28% w/v); 3.5 mM ferrous ammonium sulfate; and 16% v/v 2-propanol. In both cases, the best diffracting crystals grew in one to two weeks. The citrate-buffered crystals were typically 0.3 mm×0.1 mm×0.1 mm and belonged to the space group type *R*3 with cell dimensions *a* = 36.5 Å, *c* = 107.0 Å for the triply primitive hexagonal cell. Typical MES-buffered crystals were 0.3 mm×0.2 mm×0.2 mm, and belong to the same space group with similar cell dimensions, *a* = 134.6 Å, *c* = 104.6 Å. The structure of the BPDO_B356_: biphenyl complex was obtained by adding a small amount of biphenyl powder to crystals and incubating for a period of 24 hours before harvesting.

### Diffraction Experiments

Diffraction data were collected at cryogenic conditions (∼100K) from crystals frozen in liquid nitrogen after brief incubation in a solution similar to the reservoir solution, but with the 2-propanol replaced by 20% v/v glycerol [17]. The diffraction data were indexed and reduced to averaged intensities using the HKL program suite [44]. Intensities were converted to structure factor amplitudes using programs from the CCP4 package [45]. Prior to diffraction experiments using synchrotron radiation, crystals were typically screened for quality of diffraction and the presence of twinning using Cu-Kα radiation from a Rigaku rotating anode X-ray generator equipped with mirror optics and an R-AXIS image plate area detector (Rigaku/MSC). High-resolution diffraction data used for refinement were collected at the Advanced Photon Source synchrotron (APS) using beamlines BM-14-C and SBC-19ID and are summarized in Table 3.

**Table 3:** Summary of crystallographic data.

	BPDO_B356_ Citrate Buffer		BPDO_B356_ MES Buffer	
	Structure Solution	Structure Refinement	w/o biphenyl	w/biphenyl
Wavelength (Å)	1.54	1.00	0.90	1.01
Data Range	40-2.2	50-1.5	50-1.63	50-1.58
Space Group	*R*3	*R*3	*R*3	*R*3
*a*, Å	136.5	136.6	136.2	136.4
*c*, Å	106.5	107.2	106.2	107.2
Completeness, %	99.4 (94.2)	96.7 (78.0)	98.6 (89.0)	94.76
Unique Reflections	74538	114874	91603	91502
R_sym_, %‡	4.9 (10.1)	7.1 (12.3)	9.8 (33.4)	8.7 (49.8)
Twin Fraction	0.28	0.00	0.36	0.02
*I*/σ*_I_*	50.1 (24.1)	28.2 (7.4)	6.8 (3.7)	14.3 (2.2)

Values in parentheses pertain to the outermost shell of data.

‡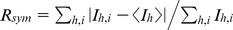.

### Detection and Analysis of Twinning

Twinning was detected by analysis of plots of the cumulative intensity distribution, *N*(*z*), [46], [47], as produced by the program TRUNCATE [48]. The comparison of observed *N*(*z*) to the expected distribution coupled with the observation of a single lattice in the diffraction pattern indicated merohedral twinning. The twin fraction was assessed by analysis of the cumulative distribution of 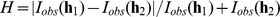, where h_1_ and h_2_ are Miller indices related by the twinning operation [49], and varied from 0–50% for the crystals used in this study as reported for each crystal in Table 3.

### Molecular Replacement and Model Building

The structure of BPDO_B356_ was determined by molecular replacement using naphthalene dioxygenase (PDB ID: 1NDO) as a search model. AMORE [50] was used to calculate the cross-rotation and translation functions. A dominant solution was obtained and used for rigid body fitting within AMORE. CNS [51] was used for further rigid body refinement and to calculate an initial map. The initial map was readily interpreted such that 534 residues (83%) of the BPDO_B356_ sequence were rapidly modeled using the program O [52].

### Refinement of crystallographic models

Initial models for the structure of the citrate-buffered crystals were refined using the program CNS with automated parameter adjustment and electron density map calculations. Final models of BPDO_B356_ and the BPDO_B356_•biphenyl complex for the structure of the MES-buffered crystals were refined using SHELX-97 [53] and REFMAC5 [54], respectively. O [52] was used for model building, electron density evaluation and superposition of models. Anomalous difference electron density maps were used to verify the presence of iron at the active site and to assess its occupancy by comparison to the density observed for iron in the Rieske cluster. Atomic models and structure factors have been deposited in the Protein Data Bank under the PDB Ids 3GZY (BPDO_B356_) and 3GZX (BPDO_B356_•biphenyl complex).

### Steady-state Kinetic and Coupling Measurements

Enzyme activity was assayed by following O_2_ consumption using a computer-interfaced Clark-type polarographic oxygen electrode essentially as described previously [17]. The standard reaction mixture contained 70 µM Fe(SO_4_)_2_(NH_4_)_2_, 288 µM biphenyl, 123 µM NADH, 1.2 µM BphG_B356_, 2.8 µM BphF_LB400_, and 0.36 µM BPDO_B356_ in air-saturated 50 mM MES buffer, pH 6.0. Initial velocity measurements were taken using concentrations ranging from 0.9–176 µM 2,2′-dichlorobiphenyl (Note: 2,2′-dichlorobiphenyl is a suspected cancer hazard and as described in the MSDS appropriate personal protection equipment and handling measures were followed). Coupling between O_2_ consumption and biphenyl turnover was estimated by adding catalase to the assay 90 s after initiating the reaction. The amount of O_2_ released was taken to reflect 50% of the hydrogen peroxide produced.

## Supporting Information

Figure S1 Sequence alignment showing low sequence identity in the region that defines the active site entrance to BPDO_B356_.
(EPS)
